# The Impact of Cerebral Amyloid Angiopathy in Various Neurodegenerative Dementia Syndromes: A Neuropathological Study

**DOI:** 10.1155/2019/7247325

**Published:** 2019-01-16

**Authors:** Jacques De Reuck

**Affiliations:** Université de Lille 2, INSERM U1171, “Degenerative & Vascular Cognitive Disorders”, CHU Lille, 59000, France

## Abstract

**Purpose:**

The Boston criteria for cerebral amyloid angiopathy (CAA) have to be confirmed by postmortem examination. The present study investigates the incidence and the cerebrovascular impact of the severity of CAA in various neurodegenerative dementia diseases.

**Material and Methods:**

208 patients underwent an autopsy. They consisted of 92 brains with Alzheimer's disease (AD), 46 with frontotemporal lobar degeneration (FTLD), 24 with progressive supranuclear palsy (PSP), 21 with Lewy body dementia (LBD), 5 with corticobasal degeneration (CBD), and 20 controls. In addition to the macroscopic examination, a whole coronal section of a cerebral hemisphere, at the level of the mamillary body, was taken for semiquantitative microscopic evaluation of the small cerebrovascular lesions.

**Results:**

CAA is present in 2/3% of the AD brains of which half of them have a severe form, grade 3. Only the latter displays more cerebrovascular lesions. CAA is present in 45% of the LBD brains. Cortical microinfarcts are only more frequent in the CAA grade 3 group. In LBD additional AD pathology is present in 41% of the CAA grade 0, 83% in grade 1-2, and 100% in grade 3. In PSP only 21% had CAA grade 1-2. In FTLD, CBD, and normal controls no CAA pathology is observed.

**Conclusions:**

The present study shows that CAA is most frequently associated to AD but that only the severe form displays more cerebrovascular lesions. LBD is the second most frequent disease associated to CAA with a clear correlation between the incidence of the associated AD features and the increasing severity of the CAA. In PSP only 21% display mild CAA features. PSP, tau-FTLD, and CBD are part of the Pick complex diseases, who are known to have a favourable vascular profile which can explain their low incidence of cerebrovascular lesions, in contrast to AD and LBD brains.

## 1. Introduction

The Boston criteria are used to suspect clinically the presence of cerebral amyloid angiopathy (CAA) [1]. It is diagnosed primarily as a cause of lobar cerebral haematoma (LCH) in the elderly. Additionally cortical superficial siderosis (CoSS), white matter changes (WMCs), cortical microinfarcts (CoMIs), and cortical microbleeds (CoMBs) are part of the pathological picture [2]. However, on neuropathological examination of a large series of intracerebral haematomas only 9.7% are found to be due to CAA [3]. In a small series of 13 patients with clinically suspected CAA the diagnosis could be confirmed by postmortem examination of the brain [4]. Overall, the incidence of CAA in the elderly varies from 31.7% up to 78,9% according to different studies [5, 6]. In an older study the incidence of CAA in Alzheimer's disease (AD) is observed in 25.6% [7]. Also, CAA is found to be associated to 50% of brains with Lewy body disease (LBD) and 25% of cases with progressive supranuclear palsy (PSP) [8].

Whether CoMIs are significantly increased in Alzheimer patients with and without CAA is still a matter of debate [9, 10].

No postmortem study has been performed about the impact of the degree of severity of CAA and its frequency in different neurodegenerative diseases.

The present observational neuropathological study investigates the incidence of the different degrees of CAA in various neurodegenerative dementia diseases and their consequences on the occurring of cerebrovascular lesions.

## 2. Material and Methods

A total number of 208 patients, who had been followed up at the Lille University Hospital, underwent an autopsy. They included 188 patients with a history of dementia and 20 controls, who died from a nonneurological disease and without a history of cognitive decline or stroke.

A previously obtained informed consent from the nearest family allowed an autopsy for diagnostic and scientific purposes. The brain tissue samples were acquired from the Lille Neuro-Bank of the Lille University, federated to the “Centre des Resources Biologiques” that acted as an institutional review board.

The standard diagnostic procedure consisted of examining samples from the primary motor cortex, the associated frontal, temporal, and parietal cortex, the primary and secondary visual cortex, the cingulate gyrus, the basal nucleus of Meynert, the amygdaloid body, the hippocampus, basal ganglia, mesencephalon, pons, medulla, and cerebellum. Slides from paraffin embedded sections were stained with haematoxylin-eosin, luxol fast blue, and Perl. Immune-staining for protein tau, *β*-amyloid, *α*-synuclein, prion protein, TDP-43, and ubiquitin was performed

On neuropathological examination 92 brains were diagnosed as AD, 46 as frontotemporal lobe disease (FTLD), 24 as PSP, 21 as LBD, and 5 as corticobasal degeneration (CBD). In the 20 controls the neurodegenerative features were absent or contained only mild AD features.

The presence of various degrees of CAA was made according the criteria of a consensus protocol and graded from 0 to 3 on examining four cortical samples with *β*-amyloid staining [11]. AD features were classified according to the Braak and Braak criteria [12]. The main diagnosis of AD was retained when stages V and VI were reached. The postmortem diagnosis of FTLD was made according to the neuropathological diagnostic and the nosological criteria of the Consortium for FTLD [13]. LBD was diagnosed according to the report of the consortium on DLB international workshop [14]. The diagnostic criteria of CBD were those proposed by the international consortium of behavioral neurology [15]. Staging of the cerebral arteriosclerotic microinfarcts (CAMIs) was performed according to the recommendations of the vascular dementia group [16]. CAMIs are presented mainly in the deep brain structures while CoMIs have another location and are due to different etiologies [17, 18].

In addition to the detection of macroscopic visible lesions such as haematomas and territorial and lacunar infarcts, a whole coronal section of a cerebral hemisphere, at the level of the mamillary body, was taken for semiquantitative microscopic evaluation of the small cerebrovascular lesions such as WMCs, CoMBs, CoMIs, and lacunes, allowing a global and more or less representative quantification of the brain lesions. The mean values of WMCs, characterized by the degree of axonal and myelin loss, were the average of the ranking scores: no change (R0), a few isolated (R1), frequently scattered in the corona radiata (R2), and forming confluent lesions (R3) of myelin and axonal loss. For the other cerebrovascular lesions their mean values corresponded to their average numbers in the individual brains.

Main age and gender distribution was also compared between the different groups.

Univariate comparisons of unpaired groups were performed with the Fisher's exact test for categorical data. The nonparametric Mann-Whitney U-test was used to compare continuous variables. The significance level, two-tailed, was set at ≤ 0.05.

## 3. Results

In AD patients 33% had CAA grade 0, 19% grade 1, 15% grade 2, and 33% grade 3. In LBD 55% had CAA grade 0, 24% grade 1, 8% grade 2, and 13% grade 3. In PSP 79% had CAA grade 0, 7% grade 1, and 14% grade 2. No grade 3 was observed in this disease entity. In FTLD, CBD, and normal controls no CAA pathology was observed (Table 1).

The age distribution and the gender distribution were not statistically different between the different neurodegenerative diseases according to their degree of CAA, varying overall from 67 up to 82 years. Male gender varied from 40 up to 67%.

Associated pathology was observed in different disease groups. In AD brains LBD pathology was present in 9% of the CAA grade 0, 16% in grade 1-2 and 9% in grade 3. CAMIs were seen in 28% of the CAA grade 0, 11% in grade 1-2, and 18% in grade 3 CAA groups. In LBD patients 41% additional grade 1-3 AD pathology was present in the CAA grade 0, 83% in grade 1-2, and 100% in grade 3. CAMIs were only observed in 17% of the grade 0 group.

In the PSP group 14% of AD pathology and 5% CAMIs were only seen in the CAA grade 0 brains. In the FTLD and control patients with only CAA grade 0, respectively, 4 and 5% mild AD pathology were observed while absent in the CBD group (Table 2).

In AD brains no statistical differences in severity of the cerebrovascular lesions were observed between CAA grade 0 and grades 1-2. However, WMCs, LCHs, CoMIs, and CoMBs were increased in grade 3 compared to the former two groups. In LBD only CoMIs were increased in the grade 3 CAA group compared to the grades 0 and 1-2. In PSP no statistical differences in cerebrovascular lesions were present between the grade 0 and grade 1-2 patients (Table 3).

## 4. Discussion

The present study shows that CAA is present in 2/3 % of the 92 AD brains of which half of them have a severe form. In AD brains only the CAA grade 3 group displays more cerebrovascular lesions than those with grades 0 and 1-2. This predominance has already been shown in a previous study, comparing AD brains with and without CAA [19]. These findings confirm that cerebrovascular lesions are more frequent in AD brains with severe CAA [20, 21]. However, CAA is mainly related to the aging process, so that other mechanisms can also contribute to the pathogenesis of CoMIs [22]. So, atherosclerotic cerebrovascular disease is the main cause of cortical cerebellar microbleeds and microinfarcts, rather than CAA [23]. Also the cerebrovascular lesions observed in AD-CAA brains are less severe than in CAA cases with lobar haematomas and without AD features [24].

LBD is the second neurodegenerative disease associated to CAA. There is a clear correlation between the frequency of AD features and the increasing severity of the CAA. This correlation between the AD and CAA features in LBD has already been previously described [25, 26]. They are responsible for an increase of CoMIs [27].

CAA is rarely associated to PSP [28]. In the present study CAA features are observed in 21%, but only in the mild forms.

PSP, tau-FTLD, and CBD are part of the Pick complex diseases, which are known to have a low incidence of cerebrovascular lesions due to a favourable vascular profile [29]. An inverted region on chromosome 17 is linked to many Pick complex diseases [30]. Presently, the low incidence or the absence of CAA and CAMIs in these disease entities confirms these findings and their differences with the other neurodegenerative diseases such as AD and LBD.

## Figures and Tables

**Table 1:** Average age (standard deviation), gender distribution and % frequency of cerebral amyloid angiopathy (CAA) according to the grading of its severity in different neurodegenerative dementia diseases.

Items	Age	Gender	CAA
	(years)	(% male)	(%)
*Alzheimer's disease (n = 92)*			
CAA			
grade 0	76 (11)	58%	33%
grade 1	76 (10)	56%	19%
grade 2	74 (10)	50%	15%
grade 3	78 (9)	41%	33%
*Lewy body disease (n = 21)*			
CAA			
grade 0	81 (7)	67%	55%
grade 1	76 (10)	60%	24%
grade 2	82 (9)	62%	8%
grade 3	82 (9)	67%	13%
*Progressive supranuclear palsy (n = 24)*			
CAA			
grade 0	74 (9)	40%	79%
grade 1	75 (10)	75%	7%
grade 2	83 (10)	75%	14%
*Frontotemporal lobar degeneration (n = 46)*			
CAA grade 0	67 (11)	53%	100%
*Corticobasal degeneration (n = 5)*			
CAA grade 0	73 (5)	40%	100%
*Normal controls (n = 20)*			
CAA grade 0	66 (12)	58%	100%

**Table 2:** Frequency of additional Alzheimer's pathology (AP), Lewy body pathology (LBP), progressive supranuclear palsy pathology (PSP), and cerebral arteriosclerotic micro-infarcts (CAMIs) in different neurodegenerative dementia diseases according to the grading of the severity amyloid angiopathy (CAA).

Items	AP	LBP	CAMIs
*Alzheimer's disease (n = 92)*			
CAA			
grade 0		9%	28%
grade 1-2		16%	11%
grade 3		9%	18%
*Lewy body disease (n = 21)*			
CAA			
grade 0	4I%		17%
grade 1-2	83%		0%
grade 3	100%		0%
*Progressive supranuclear palsy (n = 24)*			
CAA			
grade 0	14%	0%	5%
grade 1-2	0%	0%	0%
*Frontotemporal lobar degeneration (n = 46)*			
CAA grade 0	4%	0%	0%
*Corticobasal degeneration (n = 5)*			
CAA grade 0	0%	0%	0%
*Normal controls (n = 20)*			
CAA grade 0	5%	0%	0%

**Table 3:** Semiquantitative analysis of the severity of white matter changes (WMCs), lacunar infarcts (LIs), territorial infarcts (TIs), cerebral lobar haematomas (CLHs), cortical microinfarcts (CoMIs), cortical microbleeds (CoMBs) in Alzheimer's disease (AD), Lewy body disease (LBD), and progressive supranuclear palsy (PSP) according to the grading of the severity of cerebral amyloid angiopathy (CAA).

Items	WMCs	LIs	TIs	LCHs	CoMIs	CoMBs
*AD-CAA grading*						
0:	0.9 (1.0)	0.5 (1.0)	0.2 (0.5)	0.1 (0.3)	0.4 (0.9)	0.9 (0.9)
1-2:	0.9 (0.9 )	0.1 (0.5)	0.1 (0.3)	0.1 (0.3)	0.6 (1.2)	1.1 (0.8)
3:	1.6 (0.8)*∗*	0.3 (0.7)	0.4 (0.8)	0.5 (0.7)*∗*	2.2 (1.3)*∗*	1.8 (07)*∗*
*LBD-CAA grading*						
0:	0.8 (1.1)	0.1 (0.3)	0.1 (0.3)	0.2 (0.6)	0.6 (1.1)	0.0 (0.0)
1-2:	0.6 (0.7)	0.1 (0.3)	0.0 (0.0)	0.0 (0.0)	0.4 (1.3)	0.5 (0.4)
3:	0.6 (0.7)	0.1 (0.3)	0.0 (0.0)	0.0 (0.0)	1.2 (0.8)*∗*	0.3 (0.2)
*PSP-CAA grading*						
0:	1.0 (1.0)	0.1 (0.2)	0.2 (0.5)	0.1 (0.2)	0.3 (0.6)	1.0 (1.0)
1-2:	1.8 (1.3)	0.8 (1.5)	0.0 (0.0)	0.0 (0.0)	1.0 (1.4)	0.8 (1.5)

*∗* p ≤0.05.

## Data Availability

The datasets generated during and/or analysed during the current study are available from the corresponding author upon reasonable request.
